# Role of the stromal and immune microenvironment in intrahepatic cholangiocarcinoma^☆^

**DOI:** 10.1016/j.jhepr.2026.101850

**Published:** 2026-04-04

**Authors:** Silvia Affo, Daniela Sia

**Affiliations:** 1 Tumor Microenvironment Plasticity and Heterogeneity Research Group, Institut d'Investigacions Biomediques August Pi i Sunyer (IDIBAPS), Barcelona, Spain; 2 Division of Liver Diseases, Department of Medicine, Tisch Cancer Institute, Liver Cancer Program, Icahn School of Medicine at Mount Sinai, New York, New York, USA

**Keywords:** intrahepatic cholangiocarcinoma, tumor microenvironment, cancer-associated fibroblasts, Participation, immune cells, therapies

## Abstract

Intrahepatic cholangiocarcinoma (iCCA) is one of the deadliest malignancies, with an overall 5-year survival rate of approximately 10%. For decades, surgery and chemotherapy have represented the only treatment options for early- and late-stage disease, respectively. More recently, characterisation of the genomic landscape of iCCA has identified several “druggable” oncogenic drivers and led to the FDA approval of the first targeted therapies, including FGFR and IDH inhibitors, for second-line treatment in genetically defined patient subsets. Nonetheless, most patients treated with these therapies rapidly develop resistance and eventually experience disease progression. At a time when immune checkpoint inhibitors (ICIs) are profoundly reshaping cancer treatment, their use in combination with chemotherapy has yielded only modest survival benefits in iCCA, with more than two-thirds of patients exhibiting intrinsic resistance. The aggressive and refractory nature of this cancer is often attributed to its intricate tumour microenvironment (TME); however, the complex interplay between tumour cells and other TME components (*i.e*. immune cells, cancer-associated fibroblasts, and endothelial cells), as well as the molecular and cellular mechanisms driving tumour progression and therapeutic resistance, remain poorly understood. In this review, we discuss the critical role of the stromal and immune TME in iCCA and how its characterisation has informed patient stratification and molecular classification. We also describe recent findings supporting distinct genotype–immunophenotype relationships in iCCA, as well as the existence of functionally heterogeneous subsets of cancer-associated fibroblasts. Ultimately, this review aims to provide a comprehensive overview of current knowledge while stimulating discussion on therapeutic implications and future research directions.

## Graphical abstract

**Figure:**
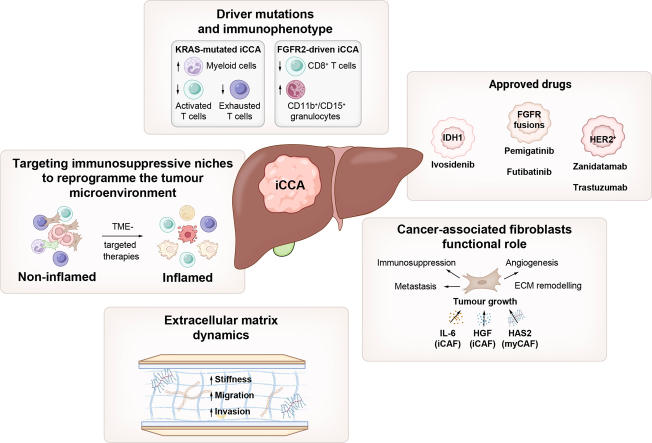

**Keypoints**

• iCCA presents with an immune cold TME characterised by poor immunogenicity and low mutational burden, limiting responsiveness to current immunotherapies.
• Emerging evidence suggests a key role of driver genetic alterations in shaping the immunophenotype of this malignancy, with *KRAS*-mutated iCCA characterised by abundance of myeloid cells, T-cell exhaustion and paucity of activated CD8^+^ T cells in both humans and mice, and *FGFR2*-driven iCCA associated with paucity of CD8^+^ T cells and abundance of CD11b^+^/CD15^+^ granulocytes.
• CAFs are key drivers of iCCA progression, orchestrating tumour growth, immune escape, angiogenesis, and extracellular matrix remodelling through mediator-driven interactions within the TME.
• Despite transcriptomic heterogeneity across CAF subtypes, their pro-tumourigenic activity appears to rely on specific mediators: iCAFs promote tumour growth through HGF and IL-6, while myofibroblastic CAFs act through HAS2.
• The extracellular matrix is not an inert scaffold but a dynamic, reactive compartment that actively drives iCCA progression through stiffness, migration, invasion, and immune modulation.
• Targeting niches rather than single cell populations represents a promising approach to reprogramme the immunosuppressive TME in iCCA.

## Introduction

Primary liver cancer represents the third leading cause of cancer-related death globally.^1^^,^^2^ The second most common primary liver cancer (∼15%-20%) after hepatocellular carcinoma is intrahepatic cholangiocarcinoma (iCCA), an aggressive malignancy of the small bile ducts within the liver.^3^^,^^4^ Based on the anatomical location, iCCA is often grouped together with other biliary tract cancers (BTC) including extrahepatic forms of cholangiocarcinoma (CCA) – perihilar and distal – and gallbladder cancer. However, it is well accepted that crucial differences exist among the distinct BTCs and between the CCA subtypes both in terms of genetic landscape,^5^ potential cell-of-origin,^6^ and management,^3^^,^^4^^,^^7^ suggesting that they represent distinct molecular and clinical entities. Here, we will focus on understanding the tumour microenvironment (TME) of iCCA.

With an incidence of 1–2 new cases per 100,000 persons per year in the US, iCCA is a relatively rare but highly fatal malignancy, accounting for less than 3% of all gastrointestinal cancers yet nearly 20% of related deaths.^3^ Despite its low prevalence, a steady increase in its incidence^8^ and mortality^9^ rates has been recorded over recent decades. In the US alone, the incidence of iCCA increased by 128% between 1973 and 2012^10^ and continues to rise.^11^ This steady increase, combined with high mortality rates,^9^ underscores the fact that optimal management of iCCA remains a challenge. Importantly, while epidemiological studies have reported increasing incidence and mortality rates for iCCA, rates for extrahepatic CCA have been steady or decreasing.^6^ Although rare in the Western world, iCCA is the most common liver cancer in Asia, particularly in Thailand, largely due to the high prevalence of risk factors such as liver fluke infection, which is endemic in the region and a well-established driver of iCCA, with incidence rates reaching up to 80 per 100,000 people per year.^3^^,^^4^^,^^7^^,^^12^ In regions where liver flukes are not endemic, other risk factors, including hepatitis B and C viruses, primary sclerosing cholangitis, obesity, diabetes II, metabolic disease and alcohol are likely contributors.^3^^,^^7^^,^^13^

Currently, the only curative option for iCCA is to diagnose the tumour early enough to enable surgical resection. However, due to mild and non-specific symptoms, more than 70% of patients are diagnosed with unresectable or metastatic disease. For advanced stage iCCA, standard chemotherapy with gemcitabine plus cisplatin has been the only *first-line* treatment since the ABC-01 trial in 2010, with a median overall survival (OS) of just 12 months.^14^ While immune checkpoint inhibitors (ICIs) as single agents have been ineffective in iCCA,^15^ recently the addition of anti-PD-L1 or anti-PD1 blockade to standard chemotherapy became the new first-line treatment option^16^^,^^17^ thanks to the success of the TOPAZ-1 and KEYNOTE-966 trials. Despite this exciting progress, only one-third of patients respond and the benefit remains quite modest, with a median overall survival of 13 months.^16^^,^^17^ For the majority of patients progressing on first-line treatment, only a few targeted therapies have been approved in second-line for small genetically defined subsets of patients, including: i) the FGFR inhibitors, pemigatinib and futibatinib, for patients harbouring *FGFR2* fusions and/or mutations; ii) the IDH1 inhibitor, ivosidenib, for *IDH1*-mutant iCCAs; iii) the monoclonal antibodies zanidatamab and trastuzumab for HER2^+^ patients; and iv) the combination of dabrafenib and trametinib for BRAF^V600E^-mutant biliary tract cancers.[18], [19], [20], [21] Although the discovery of such alterations and FDA approval of the corresponding targeted therapies have paved the way for precision medicine in iCCA, the quick onset of intrinsic or acquired resistance substantially hinders the successful treatment of this malignancy. In this scenario, understanding the intricate stromal and immune milieu of iCCA is critical to improve responses to the current treatment strategies, overcome resistance as well as design more effective strategies targeting alternative immune checkpoints.

Over recent decades, next-generation sequencing and more recently single-cell based and spatial omics have revolutionised our understanding of tumour and microenvironment heterogeneity. In iCCA, this approach has uncovered a striking level of complexity within the tumour ecosystem, revealing multiple cell types with unique and heterogeneous transcriptional profiles within the same and different cell lineages. Furthermore, these technologies enable the localisation of cell states and interactions within their tissue architecture. Because the microenvironment can exert either pro-tumourigenic or anti-tumourigenic effects depending on disease stage and patient-specific factors,^22^ understanding the determinants of these divergent states is critical for designing personalised strategies in iCCA.

Here, we discuss current knowledge on the characteristics and roles played by the immune and stromal TME components in driving growth, progression and therapeutic resistance in this fatal malignancy. Furthermore, we will discuss how the application of state-of the-art single-cell omics (extensively reviewed in ref^23^) has contributed to our understanding of the iCCA ecosystem, while outlining current limitations and future directions.

## Immune TME in iCCA: Key features and functions

### Overview of the immune TME of iCCA

**Fig. 1:**
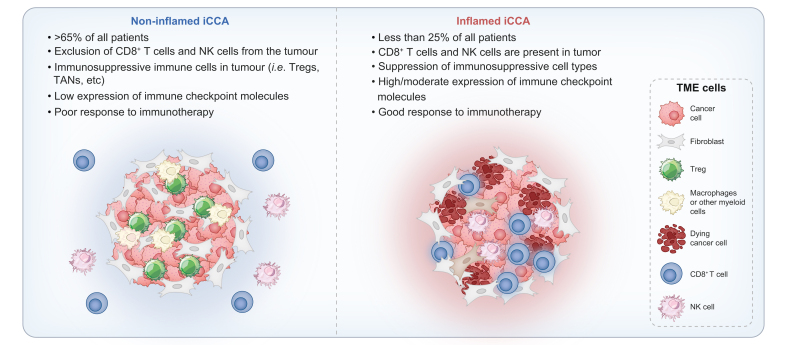
The TME of iCCA. Schematic summary of the non-inflamed (left) and inflamed (right) immune phenotypes of iCCA. Non-inflamed iCCAs account for most cases (>65%) and are characterised by exclusion of CD8^+^ T cells and NK cells, low expression of immune checkpoint molecules and immunosuppressive mechanisms. On the other end, inflamed iCCAs feature an abundance of activated CD8^+^ T cells and NK cells, and exhibit good responses to immunotherapy. iCCA, intrahepatic cholangiocarcinoma; TANs, tumour-associated neutrophils; TME, tumour microenvironment; Tregs, regulatory T cells.

Over the past decades, the application of bulk RNA sequencing and, more recently, single-cell next-generation sequencing has provided an unprecedented molecular and cellular characterisation of iCCA. From the molecular point of view, several targetable drivers have been identified in a significant fraction of iCCA, including *KRAS* mutations (∼22%), *IDH1/2* mutations (∼15-20%), *BRAF* mutations (∼5%), *FGFR2* fusions (∼10–15%), and HER2 amplifications/overexpression (5%).^5^^,^[24], [25], [26], [27], [28], [29], [30], [31] These alterations have important implications for the clinical management of the disease, as reflected by currently approved targeted therapies (*i.e*. *FGFR2*, *IDH1* and *HER2*) and those currently under clinical investigation (*i.e*. *KRAS*^*G12D*^ or ^*G12C*^). On the other hand, the growing use of immunotherapies based on immune checkpoint inhibitors (ICIs) has drawn researchers’ attention to the tumour immune microenvironment (TIME), with several characterisation studies conducted at increasing resolution in recent years. Current evidence suggests that iCCA tumours are overall poorly immunogenic, with a low/modest mutational burden (2 mut/MB)^32^^,^^33^ and a highly heterogenous immune composition, characterised by variable, but generally limited, T-cell infiltration and enrichment of immunosuppressive programmes mediated by tumour-associated macrophages (TAMs), myeloid-derived suppressor cells (MDSCs), tumour-associated neutrophils (TANs) and regulatory T cells (Tregs) (Fig. 1). Interestingly, while great inter-tumour immune heterogeneity is evident in iCCA,^34^^,^^35^ multi-regional sequencing analysis of tumour samples collected from the same patient at different time points found a relatively similar tumour ecosystem makeup, suggesting a relatively stable TME composition over time.^36^

Over 20 T-cell subsets have been identified by single-cell RNA sequencing, including naïve, cytotoxic, and exhausted populations, among others.^36^ Expression of immune checkpoint molecules, such as PD1, CTLA4 and LAG3 remains low overall (2-4%)^37^^,^^38^ and quite heterogeneous within CD4^+^ and CD8^+^ T cells^36^^,^^38^^,^^39^ and across tumour location (centre *vs*. periphery).^38^ On the other hand, PD-L1 expression is mostly restricted to pro-tumourigenic TAMs in both human and murine iCCA.^40^ In an orthotopic mouse model of iCCA, depletion of TAMs failed to reduce tumour burden due to the compensatory accumulation of granulocytic MDSCs and only combination therapies targeting both TAMs and MDSCs improved responses to ICIs, underscoring the importance of these two immunosuppressive populations in orchestrating a cold and T-cell dysfunctional TME.^40^ A recent study revealed TAMs as the most abundant immune cell type in the iCCA TME, and the protein trefoil factor 3 secreted by malignant subclones to be responsible for inducing macrophage reprogramming into a tumour-promoting state, thereby fostering an immunosuppressive environment and accelerating tumour progression.^41^ The importance of TAMs and their prognostic role in iCCA have been further emphasised by recent single-cell transcriptome-assisted multi-omics analysis of human iCCA samples.^35^^,^^42^^,^^43^ In one of these studies, Bao *et al.* identified an APOE^+^C1QB^+^ macrophage subtype associated with chronic inflammation and worse overall patient survival.^42^ In another study, a reciprocal dedifferentiation–immunosuppression loop between iCCA tumour cells and TAMs has been proposed, supporting TAM targeting as a promising therapeutic strategy, although further investigation is needed to elucidate the underlying mechanisms of this loop.^43^ More recently, spatial analyses have uncovered a distinctive triad composed of SPP1^+^ macrophages, endothelial cells, and POSTN^+^FAP^+^ cancer-associated fibroblasts (CAFs) near proliferating tumour cells, which may shield tumour cells from immune surveillance. While endothelial cells supply nutrients, SPP1^+^ macrophages interact with tumour cells via SPP1–CD44 signalling, promoting angiogenesis and creating a pro-tumourigenic niche. This configuration establishes a positive feedback loop that supports tumour growth at the invasive front.^44^

Although TANs were elusive for many years, the advent of single-cell technologies has recently improved our understanding of their complex heterogeneity and therapeutic potential,^45^ suggesting an immunosuppressive and tumour-promoting role in many solid tumours, including iCCA.^46^ Through the single-cell analysis of 189 samples derived from 124 patients and eight mice with liver cancer, including both HCC and iCCA, Xue *et al.* identified a total of 11 TAN subsets with clear tissue separation and cancer-type preference. Interestingly, the TAN subsets were conserved between humans and mice, with an overall larger fraction of TANs identified in iCCA compared to HCC, consistent with the prominent role of immunosuppressive myeloid cells in this tumour type. Among the six TAN subsets identified specifically in tumours, three subtypes, namely the CCL4^+^, SPP1^+^ and PD-L1^+^ TANs, were found associated with a pro-tumour phenotype, mostly through recruitment of TAMs (CCL4^+^ TANs) and suppression of T-cell cytotoxicity (PD-L1^+^ TANs), and poor prognosis.^46^

In addition to immunosuppressive myeloid cells, CD4^+^ Tregs preferentially infiltrate iCCA tumours compared to their non-tumoral counterpart, where they acquire a hyperactivated and highly immunosuppressive phenotype, which is accompanied by the loss of CD8^+^ cytotoxic T-lymphocytes.^47^ Tregs may also play a crucial role in negatively shaping the functional orientation of tertiary lymphoid structures (TLSs) as it was described that their frequency in intra-tumoral TLSs significantly increases along with the increasing abundance of peritumoural TLSs.^48^ Notably, intratumoural TLSs showed a higher frequency of T follicular helper cells compared to peritumoural TLSs, suggesting a more immature and dysfunctional state for the latter and ultimately reinforcing the concept that peritumoural TLSs could sabotage the antitumour activity of intratumoural TLSs as previously suggested in HCC^49^ and other cancers.^50^ Finally, although the role of B cells in iCCA remains largely underexplored, emerging evidence suggests that B cells are enriched in adjacent tumour-free tissues where they play a crucial role in forming TLSs.^51^ On the contrary, B cells are relatively scarce within the tumour nest where they display an immature phenotype and immunosuppressive features mainly driven by IL-6 and TGF-β.^51^ A thorough understanding of the functional roles and crosstalk among immunosuppressive elements, antitumor immune cells, and tumour cells could open new avenues for therapeutic intervention.

### Immune classes of iCCA

The emerging critical role of the TME in dictating therapeutic responses and prognosis has led to the generation of several immune-based classifications. While the first immune classifications were mainly based on bulk RNA sequencing, multiplex and/or single-plex immunostaining or a combination of the two, more recent studies have applied single-cell RNA sequencing to identify immune subsets at higher resolution (Table 1). Analysis of the bulk RNA sequencing of 566 human samples using a set of signatures capturing elements of the TME and relative immune functions identified four main subtypes including: immune desert (I1), which accounted for the majority of patients (45%); immune activation (I2) characterised by infiltration of immune cells, immune checkpoint pathways and prolonged survival; myeloid rich (I3), which featured abundance of monocyte-derived cell; and mesenchymal-like (I4), rich in fibroblasts.^52^ The predominantly “cold” nature of most iCCAs was further emphasised by Carapeto *et al.*, who, based on unsupervised clustering of single-plex immunostaining data across 14 immune cell populations, stratified 110 iCCA cases into four patient subgroups, with groups 3 and 4 representing mostly cold tumours and accounting for approximately half of the cohort, consistent with the I1 cluster described above (Table 1).^38^ In another study,^53^ immune exclusion was consistently observed in half of the cohort, with 43% of the iCCA samples classified as IG2 (immune excluded), 32% as IG3 (immune activated) and 25% as IG1 (Immune-suppressive). The latter class was characterised by excessive infiltration of neutrophils and immature dendritic cells whereas the hallmark of IG2 was the relatively higher tumour-proliferative activity and tumour purity.^53^ In a more recent study, single-cell RNA sequencing of 160 samples from 124 treatment-naïve patients, including 79 HCCs, 25 iCCAs, and seven mixed cholangiocarcinoma–HCCs, identified five tumor immune microenviroment (TIME) subtypes. TIME subtype 1 (TIME-immune activation or TIME-IA) is characterised by activated myeloid and T cells, including T helper and exhausted T cells, as well as high expression of IFNG, GZMB and checkpoint molecules. TIME subtype 2 (TIME-immunosuppressive myeloid or TIME-ISM) is characterised by immunosuppressive myeloid signatures whereas TIME subtypes 3 (TIME-immunosuppressive stromal or TIME-ISS) and 4 (TIME-immune exclusion or TIME-IE) are enriched in stromal signatures, with the former enriched in CAFs whereas the latter shows predominantly endothelial and mesenchymal cells. TIME subtype 5 (TIME-immune residence or TIME-IR) is characterised by an abundance of resident cells and an overall better prognosis. Interestingly, and consistent with current knowledge, TIME 2 and TIME 3 are mostly enriched in patients with iCCA and are associated with worse survival, reflecting the predominant pro-tumour role of myeloid cells and CAFs in this malignancy^46^ (Table 1).

**Table 1:** Immunogenomic classifications of iCCA.

Reference study (cohort size)	Immune classes	TME composition	Molecular/genetic features
Job *et al.*^52^ (566 iCCA samples)	Immune desert (I1), 46% of the cohort	No immune activation	
	Immunogenic (I2), 13% of the cohort	T cells (CD4+ and CD8+), B cells, M1 macrophage	
	Myeloid rich (I3), 19% of the cohort	M2 macrophages	
	Mesenchymal-like (I4), 22% of the cohort	Innate and adaptive immunity, CAFs	TGFB signalling Integrin signalling Angiogenesis
Carapeto *et al.*^38^ (110 iCCA samples)	Group 1	Inflamed	
	Group 2	Inflamed (high CD4, CD8, etc)	Chemokine signalling
	Group 3	Non-inflamed, high PD1 and LAG3 compared to Group 4	
	Group 4	Non-inflamed	
Xue *et al.*^46^ (160 liver cancer samples, including 37 iCCAs)	TIME 1 (TIME-IA)	Immune activation with activated myeloid and T cells	
	TIME 2 (TIME-ISM) (iCCA samples)	Immune suppressive myeloid	
	TIME 3 (TIME-ISS) (iCCA samples)	Immune suppressive stromal	
	TIME 4 (TIME-IE)	Immune exclusion and abundance of endothelial/mesenchymal cells	
	TIME 5 (TIME-IR)	Immune residence (Kupffer and endothelial cell)	
Martin-Serrano *et al.*^37^ (956 iCCA samples)	Immune classical, 10% of the cohort	High immune infiltration & activation	Metabolism Type I IFN signalling *TP53* mutations
	Inflammatory stroma, 23% of the cohort	Immune and stromal infiltration, T cell exhaustion, CAFs	TGFβ/AKT/mTOR signalling *KRAS* mutations
	Hepatic stem-like, 32% of the cohort	Intermediate immune and stromal infiltration, M2 macrophages	NOTCH/YAP1 signalling *IDH1* and *BAP1* mutations, *FGFR2* fusions
	Tumour classical, 11% of the cohort	Low immune infiltration	MYC signalling Cell cycle
	Desert-like, 20% of the cohort	Low/absent immune infiltration, regulatory T cells	Mitotic spindle WNT signalling *KRAS + TP53* mutations
Lin *et al.*^53^ (255 iCCA samples)	IG1 (25% of the cohort)	Immune suppressive, high immune infiltration of neutrophils and immature DCs	*KRAS* mutations
	IG2 (43% of the cohort)	Immune exclusion	High tumour proliferation High tumour mutational burden
	IG3 (32% of the cohort)	Immune activated with adaptive immune cells, NK and activated DCs	

CAF, cancer-associated fibroblasts; FGFR2, fibroblast growth factor receptor 2; IDH1, isocitrate dehydrogenase (NADP+) 1; TGFβ, transforming growth factor beta; TME, tumour microenvironment; Tregs, regulatory T cells.

Given the complex and critical crosstalk among tumour cells and the immune and stromal components of the TME, other classification systems have adopted more integrative multi-omics approaches rather than relying solely on immune-based strategies. According to Martin-Serrano *et al.*’s classifications of ∼900 patient samples, iCCA can be divided into five TME-based classes called: immune classical, classical, inflammatory stroma, hepatic stem-like, tumour classical and desert-like^37^ (Table 1). The immune classical subtype (∼10%) is characterised by high immune infiltration and immune activation.^37^ These tumours show frequent *TP53* mutations and are predicted to respond well to ICIs. In contrast, the inflammatory stroma subtype (∼25%) exhibits immune infiltration with exhausted T cells and abundant fibrotic stroma. This group is frequently associated with *KRAS* mutations, reflecting potential mechanisms of immune evasion and resistance to ICIs as shown in *KRAS*-driven mouse models of the disease.^37^^,^^54^ The hepatic stem-like subtype (∼35%) features a low immune infiltration, a stem-like phenotype and abundance of M2 macrophages along with enrichment of *IDH1/2* mutations, *FGFR2* fusions, and *BAP1* loss. This group is responsive to targeted therapies, including IDH1 (ivosidenib) and FGFR inhibitors (pemigatinib, futibatinib).^20^^,^^37^^,^^55^^,^^56^ Finally, the tumour classical (10%) and the desert-like (20%) subtypes are enriched in *TP53* and *KRAS* mutations and defined by immune exclusion. These tumours are unlikely to benefit from immunotherapy alone and may require tailored strategies to convert them from non-inflamed into inflamed tumours. Understanding the molecular mechanisms underpinning immune evasion and immunosuppression in each of the immune classes identified thus far is key to the design of more effective therapeutic strategies.

### Emerging genotype-immunophenotype relationships in iCCA

The observed associations between key driver mutations and distinct immune profiles/subtypes suggest that the immunogenicity of iCCA may be, at least partially, influenced by unique, and potentially actionable, genotype-immunophenotype relationships (Table 2). Consistently, *KRAS* mutant iCCAs have been found to be associated with an inflamed phenotype characterised by an abundance of myeloid cells, T-cell exhaustion and paucity of activated CD8^+^ T cells in both human and murine iCCAs^37^^,^^54^^,^^57^^,^^58^ (Table 2). Whether co-occurrence of *KRAS* and *TP53* mutations confers a unique non-inflamed immunophenotype enriched with Tregs in human iCCA requires further investigation. Current preclinical data from an orthotopic mouse model carrying the same alterations do not support this hypothesis, as the model shows no immunological differences compared with other KRAS-mutant murine iCCA, suggesting potential species-specific differences^37^^,^^58^ (Table 2). Regarding the molecular mechanisms underpinning the immunoregulatory role of mutant *KRAS*, a recent study showed that overexpression of the IL1RN-201/203 splicing variants is able to remodel the TME of *KRAS*-driven murine iCCA by hampering myeloid recruitment and allowing significant accumulation of cytotoxic T cells.^54^ Using a conditional *KRAS*-mutant murine model, Qiao *et al.* demonstrated that turning off *KRAS* triggers rapid tumour regression accompanied by activation of the TGF-β pathway and cellular senescence. Senescent CCA tumour cells secrete pro-inflammatory factors, including IL-15 and CCL17, which play a crucial role in inducing immune surveillance with infiltration and enrichment of activated CD8^+^ T cells and subsequent tumour regression.^59^

**Table 2:** Most relevant genotype-immunophenotype relationships described in iCCA.

Gene	Reference study	Co-mutation profile	Host	Immune phenotype	TME composition
*KRAS*	Martin-Serrano *et al.*^37^	*KRAS*^*mut*^*(w/o TP53*^*mut*^*)*	Human	Inflamed	Increased: Myeloid cells, T-cell exhaustion, ICI resistance signature
		*KRAS*^*G12D*^*, P19 loss (w/o TP53*^*mut*^*)*	Mouse (HTVI)	Inflamed	Increased: G-MDSCs, CD8^+^PD1^+^, ICI resistance
		*KRAS*^*mut*^, *TP53*^*mut*^	Human	Non-inflamed	Increased: Tregs, ICI resistance signature Decreased: CD8^+^ T cells
	Tomlinson *et al.*^58^	*KRAS*^*mut*^*(with TP53*^*mut*^*)*	Mouse (cell line, orthotopic)	Inflamed	Increased: TAMs, M-MDSCs, T-cell exhaustion, ICI sensitivity
	Zhang *et al.*^54^	*KRAS*^*mut*^*(w/o TP53*^*mut*^*)*	Human	Inflamed	Increased: Neutrophils, immature DCs, Tregs Decreased: CD8^+^ T central memory, CD8^+^ activated T cells
		*KRAS*^*G12D*^*, YAP1, AKT1*	Mouse (HTVI)		Increased: Neutrophils via IL1RN, alternative splicing
*IDH1*	Baretti *et al.*^60^	*IDH1*^*mut*^	Human		Increased: TAMs, CAFs, proximity between CD8^+^ T cells and TAMs
	Lin *et al.*^57^	*IDH1/2*^*mut*^	Human	Inflamed	Increased: CD8^+^ T cells, cytotoxic cells
	Wu *et al.*^61^	*IDH1*^*mut*^*(with KRAS*^*mut*^*)*	Mouse	Non-inflamed	Increased: CD8^+^-cell suppression via TET, ICI resistance
*FGFR2*	Lin *et al.*^57^	*FGFR2 fusions*	Human	Non-inflamed	Decreased: CD3^+^ T cells, CD20^+^ B cells, CD15^+^ neutrophils
	Baretti *et al.*^60^	*FGFR2 fusions*	Human	Non-inflamed	Increased: CD11b^+^/CD15^+^ granulocytes Decreased: CD8^+^ T cell

CAF, cancer-associated fibroblasts; FGFR2, fibroblast growth factor receptor 2; HTVI, hydrodynamic tail vein infection; IDH1, isocitrate dehydrogenase (NADP+) 1; ICI, immune checkpoint inhibitor; M-MDSC, monocytic myeloid-derived suppressor cells; TAMs, tumour-associated macrophages; TME, tumour microenvironment; Tregs, regulatory T cells; w/o, without.

Emerging data suggest that *FGFR2* fusion-driven iCCAs exhibit a unique non-inflamed phenotype.^37^^,^^57^ Although the lack of an immunocompetent mouse model of *FGFR2* fusion-driven iCCA currently limits our understanding of the potential role of *FGFR2* fusions in shaping the immune landscape of these tumours, a clear correlation between *FGFR2* fusions, paucity of CD8^+^ T cells and abundance of CD11b^+^/CD15^+^ granulocytes has recently been identified in a small human cohort analysed by high-parameter spatial immune phenotyping.^60^ In this study, granulocytes represented more than 45% of all CD45^+^ cells in *FGFR2*-driven iCCA and showed several spatial relationships with multiple other cell types (including tumour cells, and CD4^+^ and CD8^+^ T cells).^60^ In comparison, tumours with *IDH1* mutations showed a trend toward more fibroblasts and were characterised by a closer proximity of tumour cells to CD4^+^ T cells, as well as between macrophages and multiple structural components of the TME, compared with other subtypes.^60^ While these findings require further investigation, conflicting data exist regarding whether IDH1-mutated iCCAs are associated with a non-inflamed^37^^,^^61^ or inflamed immunophenotype^57^ (Table 2). A mouse model of iCCA seems to support the notion that *IDH1-*mutant iCCA is characterised by immune evasion marked by exclusion of cytotoxic CD8^+^ T cells and that pharmacologic inhibition of mutant *IDH1* with ivosidenib reactivates CD8^+^ T cells and sensitises tumours to ICIs targeting CTLA4.^61^ Altogether, these findings suggest that understanding the mechanisms underpinning each genotype–immunophenotype relationship may enable the development of novel therapeutic strategies combining targeted therapies with immunotherapy for specific subsets of patients with iCCA.

## CAFs and other stromal cells

### CAF origin, heterogeneity, and functions in iCCA

**Fig. 2:**
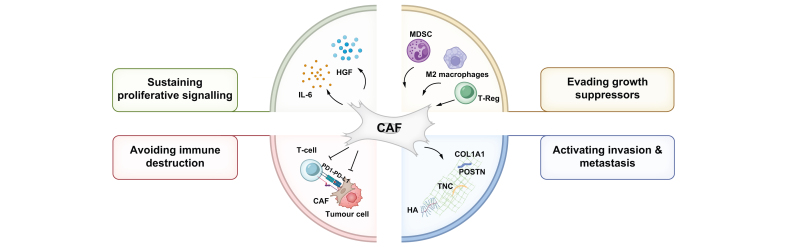
The hallmarks of CAFs in iCCA. CAFs contribute to cancer hallmarks through multiple processes: (i) secretion of growth factors and cytokines, contributing to sustained proliferative signalling; (ii) modulation of immune cell recruitment, creating an immunosuppressive milieu enriched in M2 macrophages, MDSCs, and Tregs, which help tumours evade growth suppressors; (iii) ECM remodelling, which contributes to invasion and metastasis; and (iv) induction of T-cell exhaustion via immunosuppressive mechanisms, helping tumours avoid immune destruction. COL1A1, collagen 1a; HA, hyaluronan; HGF, hepatocyte growth factor; IL-6, interleukin 6; M2, type 2 macrophage; MDSC, myeloid-derived suppressor cell; POSTN, periostin; TNC, tenascin C; Treg, regulatory T cell.

CAFs are activated fibroblasts that are abundant and widely distributed within the iCCA TME. Despite diverse cellular sources having been attributed to CAFs in liver tumours, lineage tracing studies, single-cell RNA sequencing, and ligand–receptor analyses have identified hepatic stellate cells (HSCs) as the main source of CAFs in both experimental mouse models and patients,[62], [63], [64], [65] followed by portal fibroblasts and other mesenchymal populations. The tumour-promoting role of CAFs in iCCA has been demonstrated in experimental models. Using syngeneic systems, Mertens *et al.* showed that killing CAFs in iCCA using the BH3-mimetic navitoclax reduced tumour progression.^66^ These findings were confirmed a few years later by Affo *et al.*, showing that genetic depletion of HSC-derived CAFs and αSMA^+^ CAFs in endogenously arising iCCA models reduced tumour cell proliferation and iCCA progression.^63^ Accordingly, pan-CAF gene signatures correlate with reduced overall survival in patients, highlighting the therapeutic potential of CAFs in iCCA. While the abundance of CAFs within the tumour mass has been well described, less is known about the processes that induce the activation of liver fibroblasts into CAFs. While in HCC the activation of HSCs and the onset of fibrosis represent a pre-cancerous condition that promotes cancer development,^62^^,^^65^ this is not the case in iCCA, where tumour cells appear to “educate” liver fibroblasts, inducing their activation into CAFs to support tumour growth and metabolic needs through the secretion of pro-tumourigenic and hypoxia-related factors.^65^^,^^67^ Embedded in the tumour mass, CAFs are one of the most interactive cell types in iCCA. As predicted by ligand-receptor interaction analysis of single-cell RNA-sequencing data, CAFs are one of the TME cell types most engaged in crosstalk networks, interacting with tumour cells, endothelial cells, and immune cells. CAFs are key players in the TME of iCCA, where they support tumour growth and resistance to therapy, orchestrating immunosuppression, neo-angiogenesis, invasion and metastasis, metabolic reprogramming, hypoxia, senescence, and stiffness.^3^^,^[62], [63], [64], [65]^,^^67^ CAFs contribute to cholangiocarcinogenesis through four main mechanisms: i) their secretome – including cytokines, growth factors,^62^^,^^63^^,^[68], [69], [70], [71] and extracellular vesicles[72], [73], [74] – which promotes proliferative signalling, angiogenesis, and metabolic reprogramming; ii) recruitment of immunosuppressive cells,^58^^,^^64^^,^^65^^,^[75], [76], [77] supporting immune evasion and inflammation; iii) T-cell inactivation, directly enabling immune evasion;[78], [79], [80], [81] iv) extracellular matrix (ECM) remodelling,^62^^,^^65^^,^[82], [83], [84], [85], [86] favouring tumour stiffness and promoting invasion and metastasis (Fig. 2).

**Fig. 3:**
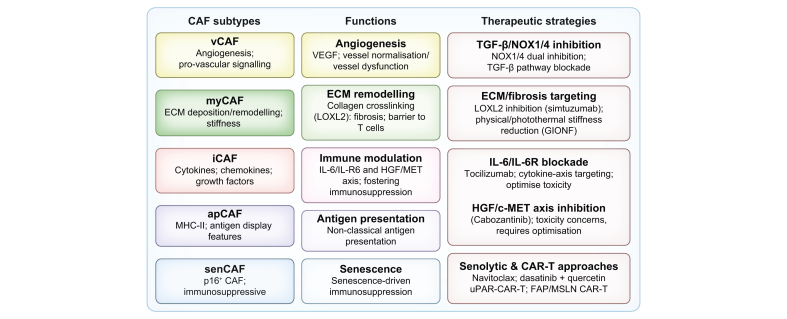
CAF subtypes, functions, and therapeutic strategies in iCCA. Heterogeneous CAF subtypes co-exist within the iCCA TME including: vascular CAFs (vCAFs), associated with angiogenesis; inflammatory CAFs (iCAFs), enriched in cytokine and immune-modulatory pathways; myofibroblastic CAFs (myCAFs), driving ECM deposition and remodelling, and stiffness; antigen-presenting CAFs (apCAFs), characterised by MHC-II expression; and senescent CAFs (senCAFs), marked by p16 expression and immunosuppressive signalling. Different therapeutic strategies targeting CAFs are under investigation. ECM, extracellular matrix; FAP, fibroblast activation protein; GIONF, gold-decorated iron oxide nanoflowers; LOXL2, lysyl oxidase-like 2; MDSC, myeloid-derived suppressor cell; MSLN, mesothelin; NOX, NADPH oxidase; TGF-β, transforming growth factor beta; Treg, regulatory T cell; uPAR, urokinase plasminogen activator receptor.

Single-cell RNA sequencing studies have also been essential in deciphering transcriptional CAF heterogeneity both in human and experimental models of iCCA. Single-cell transcriptomic analyses of iCCA^37^^,^^62^^,^^63^^,^^69^^,^^71^ have identified consistent CAF subtypes (Fig. 3), including vascular CAFs (vCAFs), associated with angiogenesis; inflammatory CAFs (iCAFs), enriched in cytokine and cytokine-receptor pathways and immune response pathways; myofibroblastic CAFs (myCAFs), enriched in ECM pathways; and antigen-presenting CAFs (apCAFs), characterised by antigen-presenting features. While immunosuppressive CAFs (isCAFs) have recently emerged as a new subtype, their functional role seems to be more related to specific mediators and their targeting seems promising. In addition, isCAF signatures are shown to be a good tool for patient stratification.[87], [88], [89] Initial studies have identified some of the mediators responsible for the CAF-driven pro-tumourigenic activity. Preclinical models have shown that HGF and IL-6 (iCAF mediators) as well as HAS2 (myCAF mediator) can promote tumour growth.^63^^,^^71^ Conversely, the myCAF marker collagen 1a1 does not influence tumour growth in preclinical iCCA models.^63^ Therefore, although transcriptomic CAF signatures may help stratify patients, their functional relevance should be validated by interrogating specific mediators – or their combinations – in preclinical models. While for other tumours such as pancreatic cancer and liver metastases, CAFs with tumour-restrictive properties have been identified,[90], [91], [92] this is not yet the case in iCCA, where despite transcriptomic heterogeneity, CAFs have only been shown to exert tumour-promoting functions. Accordingly, enrichment in pan-CAF gene signatures was associated with reduced overall survival in patients with iCCA.^63^^,^^67^

### Role of the ECM in iCCA

The ECM, mainly produced by CAFs, is the acellular component of the TME, historically considered an inert structural part of the tumour mass. Its role was primarily associated with acting as a physical barrier that traps immune cells within tumours and contributes to tissue stiffness – features linked to poor prognosis and reduced patient survival.^84^^,^[93], [94], [95], [96] In line with these functions, therapeutic strategies targeting ECM remodelling have been extensively explored, including enzymatic degradation, integrin blockade, and inhibition of crosslinking within the matrix. Recent evidence highlights the promise of these approaches for improving treatment outcomes in desmoplastic tumours.^67^

CAFs are the principal producers of ECM. A comprehensive characterisation of ECM composition across stages of cholangiocarcinogenesis remains an unmet need, particularly to elucidate whether, and how, CAF plasticity is coupled to ECM remodelling in iCCA. Addressing this will be critical for the rational design of therapies aimed at modulating the ECM to enhance treatment response. Far from being an inert scaffold, the ECM constitutes an active and reactive compartment of the tumour mass.^80^ It participates in multiple pathophysiological processes that drive iCCA progression, including stiffness, migration, invasion, and immune modulation. In iCCA, the ECM is dynamic and undergoes continuous remodelling and regulated degradation of matricellular proteins – among them collagens, periostin, osteopontin, and tenascin-C.^3^^,^^65^^,^^82^^,^[97], [98], [99]

Imbalance in ECM remodelling not only sustains tumour growth but also reshapes key tumour properties such as mechanical stiffness, cell motility, and immune-cell recruitment. For example, periostin, by interacting with integrin α5β1, activates PI3K/AKT signalling to promote tumour proliferation and invasion.^100^ Periostin also fosters immunosuppression via recruitment of TAMs and by promoting epithelial–mesenchymal transition,^99^^,^^101^ contributing to an immunosuppressive TME. Moreover, periostin interacts with collagen I and tenascin-C, amplifying tumour growth.^99^ Tenascin-C participates in tumour remodelling by activating Wnt/MAPK signalling in tumour cells and by stimulating CAFs to secrete pro-angiogenic factors.^80^^,^^83^ Osteopontin, expressed by both tumour and stromal compartments in iCCA, functions both as a cytokine and a matricellular protein; and sustains tumour growth and metastasis through MAPK1 signalling and Wnt/β-catenin activation.^102^ Notably, osteopontin is also overexpressed in the stroma of patients with iCCA and metabolic syndrome, suggesting a potential role as a driver of cholangiocarcinogenesis in this context.^103^

Collagens – especially COL1A1 – have been extensively studied in different tumours, including iCCA. While COL1A1 is a major inducer of mechanosignalling and stiffness,^62^^,^^63^ pivotal for tumour initiation and progression,[104], [105], [106] its abundance does not correlate directly with tumour growth in iCCA.^62^^,^^63^ Whether COL1A1 targeting can beneficially remodel the TIME in iCCA remains unresolved and warrants investigation as a potential avenue for combination therapies.

Strategies to manipulate collagen in iCCA include inhibition of lysyl oxidases (LOX family), enzymes that catalyse collagen crosslinking. LOX activity contributes to iCCA development and progression, and LOX expression correlates with poor prognosis.^85^^,^^86^ Recent evidence indicates that LOX remodels the TME, upregulating oxidative phosphorylation and enhancing stemness and motility.^86^ In line with these findings, pan-LOX inhibition relieves mechanical compression of tumour vasculature, enhancing cytotoxic efficacy. When combined with chemotherapy, LOX targeting promotes the release of damage-associated molecular patterns and is associated with better anti-tumour T-cell responses.^85^

Among non-collagenous ECM components, hyaluronic acid (HA) – predominantly synthesised by HAS2 in the liver – has been shown to promote tumour growth in iCCA.^63^^,^^107^ Interestingly, in experimental iCCA models, *Has2* deletion in HSC-derived CAFs did not alter tumour stiffness but markedly reduced HA content and tumour cell proliferation, supporting HAS2 as a candidate for combination therapies.^63^ Given the detrimental clinical outcomes associated with the hyaluronidase PEGPH20 in metastatic pancreatic adenocarcinoma,^108^ targeting HAS2 expression appears a safer and potentially more effective strategy to be explored in iCCA, rather than directly targeting HA.

### Tumour-associated endothelial cells

Beyond immune cells and CAFs, tumour-associated endothelial cells (TECs) add complexity to the iCCA TME. TECs drive angiogenesis and lymphangiogenesis, processes essential for tumour growth and metastasis. While most iCCAs exhibit a fibrotic, hypovascular stroma,^3^^,^^7^^,^^62^^,^^80^ tumours arising in cirrhotic or pre-cirrhotic livers often show hypervascular enhancement, resembling HCC.^109^ Notably, hypervascular iCCA has recently been shown to correlate with better immune infiltration and prognosis compared to hypovascular tumours.^110^

Lymphangiogenesis facilitates nodal and distant metastasis and is associated with recurrence and poor survival in iCCA.^80^^,^^111^^,^^112^ Single-cell RNA sequencing recently revealed the presence of five TEC subpopulations with distinct spatial and functional profiles in iCCA: MGP^+^ Endo, FCGR2B^+^ Endo – present in non-tumour tissues and related to inflammatory pathways; NPIPB4^+^Endo, IGLC3^+^Endo – found in lymphatic tissues, and related to lymphocyte activation; PDPN^+^ Endo – prevalent in tumour tissues, and related to ECM remodelling and angiogenesis.^113^ A TEC score derived from these signatures predicted prognosis and immunotherapy response. Moreover, high TEC scores correlated with *KRAS* mutations, neutrophil infiltration, and reduced NK/CD8^+^ T cells, indicating an immunosuppressive phenotype, while *BAP1* mutations and *CXCL12* overexpression showed opposite trends. Spatial transcriptomics suggested TEC–immune interactions via the CXCL12/CXCR4 axis.^113^

TECs also interact with TAMs and CAFs, promoting angiogenesis through VEGF-C/D–VEGFR-2/3–NRP-2 signalling, and activating PKC–ERK1/2 and AKT pathways.^68^^,^^80^^,^^111^ Additional mediators include CCL21/CCR7, FGFs, PDGFs, and angiopoietins.^111^^,^^114^ Tumour hypoxia amplifies these signals via HIF-1α, increasing VEGF-C and lymphatic vessel density, which correlates with poor survival in these patients.^97^^,^^112^^,^^115^^,^^116^

## Therapeutic implications

### Overcoming immunotherapy resistance

Immunotherapy with ICIs has become a mainstay of the treatment of advanced iCCA as first-line standard of care in combination with standard chemotherapy. However, the majority of patients (>70%) are intrinsically resistant, underscoring the need to design more effective combinations as well as identify biomarkers for better patient stratification. Towards these objectives, we expect that the application of single-cell RNA sequencing and spatial transcriptomics in clinical specimens paired with mechanistic studies in clinically relevant preclinical models will be crucial. As an example, growing understanding of the immune repertoire of genetically defined iCCA subsets such as *FGFR2* fusion-driven iCCAs has identified the accumulation of CD11b^+^/CD15^+^ granulocytes^60^ as potential therapeutic vulnerabilities. Future studies investigating the pro-tumourigenic role and therapeutic potential of these emerging immune subsets are expected. Another study found that tumour-derived CD109 is closely associated with an immunosuppressive TME characterised by accumulation of CD73^+^ macrophages, ultimately hindering T-cell immune responses.^117^ Whether dual blockade of CD109 and PD-L1 could improve antitumour immune responses and prolong patient survival needs to be elucidated. Novel immunotherapeutic strategies may also include targeted therapies (*i.e*. *KRAS* inhibitors) and combination treatments targeting both immune exhaustion and the surrounding TME. Similarly, increasing efforts aimed at identifying alternative immune checkpoint targets (*i.e*, B7-H3, B7-H4, *etc*.) are already underway. In this regard, several clinical trials are currently testing therapeutics targeting B7-H4 including CART T-cell and antibody-drug conjugates (NCT05180474, NCT06126666). Finally, the addition of a second ICI to the standard of care represents another option. In this respect, the addition of anti-CTLA4 therapy to anti-PD1 and gemcitabine/cisplatin has recently shown substantial survival benefits in immunotherapy-resistant orthotopic murine models of iCCA. These effects were associated with an increased abundance of activated CD8^+^CXCR3^+^IFNγ^+^ T cells, suggesting that this combination strategy warrants clinical evaluation to overcome treatment resistance in patients with iCCA.^118^

### Therapeutic implications of CAF heterogeneity

Despite the overall tumour-promoting role of CAFs in iCCA, the specific role of unique mediators remains to be elucidated. Advanced technologies such as spatial transcriptomics and multi-omics offer valuable opportunities to better understand the role of specific CAF subtypes based on their transcriptomic profiles, spatial context, and interactions with other cell types, thereby enabling the development of novel therapeutic strategies. Despite several attempts to therapeutically target CAFs, including clinical trials using TGF-β–blocking antibodies such as fresolimumab and metelimumab, these strategies have failed,^65^ and no FDA- or EMA-approved therapies currently exist for CAF targeting. Among proposed approaches, inhibition of TGF-β signalling remains one of the most appealing strategies. In their recent study, Amengual *et al.* showed how dual inhibition of NADPH oxidase 4 (NOX4) and NOX1, downstream mediators of TGF-β signalling expressed by CAFs, significantly reduced tumour growth *in vitro* and in experimental iCCA models.^81^

CAF heterogeneity underscores the need for therapies directed at functionally distinct subsets. Senolytic strategies targeting senescent CAFs have shown promise, with agents such as navitoclax or the combination of dasatinib and quercetin capable of clearing p16^+^ senescent CAFs and reducing immunosuppression, thereby impacting tumour progression.^66^^,^^119^^,^^120^ Additionally, chimeric antigen receptor-T (CAR-T) cells engineered to target the senescence-associated protein urokinase plasminogen activator receptor (uPAR) have successfully depleted senescent HSCs and attenuated liver fibrosis in experimental murine models of metabolic dysfunction-associated steatohepatitis,^121^^,^^122^ suggesting that senescent HSC-derived CAF targeting may represent a promising future approach for liver cancer.

As previously discussed, ECM remodelling is another attractive therapeutic avenue for CAF targeting. Inhibition of collagen crosslinking mediated by LOXL2 using simtuzumab has recently been shown to enhance T-cell migration and reduce tumour growth.^86^ Physical and photothermal strategies aimed at reducing tumour stiffness through CAF depletion have also demonstrated therapeutic potential, particularly through the use of multifunctional iron oxide nanoflowers decorated with gold nanoparticles, which induced tumour stiffness reduction and tumour regression.^123^^,^^124^

Therapeutic approaches directed at well-established tumour-promoting CAF mediators include IL-6/IL-6R axis inhibition with agents such as tocilizumab;^71^^,^^125^^,^^126^ and blockade of the HGF/c-MET axis using cabozantinib. However, these strategies are associated with severe toxicity and require further optimisation.^63^^,^^127^^,^^128^ Combination strategies involving inhibition of specific mediators together with chemotherapy or ICIs should also be evaluated in preclinical models, as they may enhance immune cell recruitment and activity. Extensive efforts have focused on immunotherapeutic strategies, including CAR-T cell therapy, cancer vaccines, and antibody-based approaches. Despite these advances, the therapeutic potential of CAR-T cells and vaccines for cholangiocarcinoma and its TME remain largely underexplored. Among the most promising CAR-T cell strategies against CAFs in iCCA are those targeting fibroblast activation protein and mesothelin, which have demonstrated the ability to reduce ECM deposition, improve T-cell infiltration, and inhibit tumour growth in preclinical models of other cancers with similar desmoplastic features.[129], [130], [131], [132], [133], [134]

### Future directions: multi-omics and AI-driven strategies for TME targeting

Given the complexity of the TME in iCCA, the development of combined therapeutic approaches represents a promising path toward more effective treatments, aimed at targeting not only tumour cells, but also stromal cells and their interactions. Toward this goal, the advent of spatial transcriptomics, spatial proteomics, and artificial intelligence (AI)-driven digital pathology has ushered in a new era. These approaches not only enable visualisation of cellular communities and their interactions *in situ*, but are also rapidly emerging as powerful predictive tools that link spatial organisation with clinical parameters to predict prognosis and therapeutic response. The integration of multi-omics data is expected to drive the design of novel drugs that focus on tumour cell communities and ecological niches rather than individual cell types, ultimately perturbing the entire tumour ecosystem. As an example, the recent integration of multiplexed immunohistochemistry, high-dimensional cytometry and single-cell technologies revealed key cellular cross-talk driven by stromal cells and macrophages in patients with poor prognosis.^135^ A deep understanding of these interactions is needed to improve therapeutic options. Furthermore, the convergence of spatial technologies with histology and single-cell sequencing, empowered by AI, will enable predictive modelling for rational tissue architecture reprogramming. In iCCA, the first spatial transcriptomics datasets – beginning in 2024 – have begun mapping stromal and immune cell distributions, tumour niches, and intercellular interactions. Although current cohorts remain small due to the high cost of these technologies, spatial transcriptomics provides unprecedented resolution for neighbour analysis, enabling the study of real physical proximity and interaction networks rather than purely inferred predictions. A deep-learning system trained on spatial TME features can now predict patient prognosis from a single 1-mm^2^ tumour sample with remarkable accuracy,^136^ offering a foundation for precise patient classification and personalised treatment planning in iCCA. Early translational efforts have combined digital pathology, whole-transcriptome profiling of macro-dissected tumour regions, and targeted digital spatial profiling of tumour-infiltrating myeloid cells to stratify patients into rapid progressors (<6 month survival) and long survivors (>23 months) in a cohort of patients with advanced iCCA with similar baseline characteristics, but markedly divergent outcomes following chemotherapy.^137^ These findings underscore the potential of spatially resolved biomarkers to guide treatment decisions and highlight the importance of viewing tumours as complex ecosystems where therapeutic success depends on understanding the spatial organisation of cellular communities’ and not just single cells.

However, both multi-omics technologies and AI face several limitations. Some recently developed spatial methods capture specific regions using selected markers, others cover the whole transcriptome but at lower spatial resolution, and single-cell approaches often rely on customised or commercially available panels covering up to 5,000–6,000 genes. More importantly, the volume and complexity of data generated from multi-omics studies often outstrip the capabilities of traditional AI methods. The continuous development of more advanced methods for complex data integration, along with more functional approaches able to capture changes over time and not only snapshots of the TME, is needed to translate these new technologies into real diagnostic and prognostic tools and for the development of novel therapeutic strategies. To overcome these challenges, integration with imaging technologies, validation in larger clinical cohorts and validation through functional experimental models and mechanistic studies remains essential to confirm biological relevance. Careful selection of technology platforms tailored to specific biological questions is also critical to maximise biological insight and ensure clinical translation.

## Conclusions

Currently, the complex heterogeneity of iCCA both at the molecular and cellular level represents one of the major obstacles to the successful development of more effective clinical regimens. This holds true particularly when compared to other primary liver cancers, such as hepatocellular carcinoma, where immunotherapeutic combinations have radically transformed management. To improve responses and overcome current limitations, it remains crucial to gain a deeper understanding of the dynamic relationships between tumour cells, immune cells, CAFs and treatment response. While it is certain that more studies are needed, a more systemic analysis of patient samples, particularly for those progressing or primary resistant to the standard of care, holds promise to unveil novel TME-related targets and inform future trial development. With the advent of AI and single-cell technologies, more efforts in this direction are eagerly awaited. Certainly, existing preclinical and clinical data point towards the need for combinatorial approaches rather than single agent-based strategies. Furthermore, given the complexity and heterogeneity of the TME of iCCA, we firmly believe that combination strategies simultaneously targeting more than one cell compartment or aiming at blocking crucial tumour-TME interactions within the iCCA ecosystem hold a great deal of therapeutic potential.

## Abbreviations

AI, artificial intelligence; apCAFs, antigen-presenting cancer-associated fibroblasts; BTC, biliary tract cancer; CAFs, cancer-associated fibroblasts; CAR-T, chimeric antigen receptor T-cell; CCA, cholangiocarcinoma; ECM, extracellular matrix; HA, hyaluronic acid; HCC, hepatocellular carcinoma; HSCs, hepatic stellate cells; iCAFs, inflammatory cancer-associated fibroblasts; ICIs, immune checkpoint inhibitors; iCCA, Intrahepatic cholangiocarcinoma; isCAFs, Immunosuppressive cancer-associated fibroblasts; LOX, lysyl oxidase; MDSCs, myeloid-derived suppressor cells; myCAFs, myofibroblastic cancer-associated fibroblasts; TAMs, tumour-associated macrophages; TANs, tumour-associated neutrophils; TECs, tumour-associated endothelial cells; TIME, tumour immune microenvironment; TLSs, tertiary lymphoid structures; TME, tumour microenvironment; Tregs, regulatory T cells; uPAR, urokinase plasminogen activator receptor; vCAFs, vascular cancer-associated fibroblasts.

## Authors’ contributions

Conception and design, writing of original draft, critical revision of all content and final approval of the article: Sia D and Affo S.

## Financial support

DS is supported by the 10.13039/100000005Department of Defense (RA220126), 10.13039/100000054National Cancer Institute (NCI, R01-CA285580-01) and supported by RSG-23-1151048-01-IBCD from the 10.13039/100000048American Cancer Society, [ACS.RSG-23-1151048-01-IBCD.pc.gr.175471" title="https://doi.org/10.53354/ACS.RSG-23-1151048-01-IBCD.pc.gr.175471">https://doi.org/10.53354/ACS.RSG-23-1151048-01-IBCD.pc.gr.175471]. SA is supported by 10.13039/501100018779Fero Foundation, the Spanish National Health Institute MCIN/AEI/ 10.13039/501100011033 and 10.13039/501100002924FEDER (PID2024-160007OB-I00); The 10.13039/501100000781European Research Council, grant agreement 101077312; and Ramon y Cajal program (RYC2022-036321-I). This review is based upon work from 10.13039/501100000921COST Action Precision-BTC Network, CA22125, supported by COST (10.13039/501100000921European Cooperation in Science and Technology). Figures were made with the use of BioRender.

## Conflict of interest

The authors declare no conflict of interest relevant to this work.

Please refer to the accompanying ICMJE disclosure forms for further details.
